# Caught Between Autonomy and Insecurity: A Work-Psychological View on Resources and Strain of Small Business Owners in Germany

**DOI:** 10.3389/fpsyg.2020.525613

**Published:** 2020-10-15

**Authors:** Kathleen Otto, Martin Mabunda Baluku, Lena Hünefeld, Maria U. Kottwitz

**Affiliations:** ^1^Department of Work and Organisational Psychology, Faculty of Psychology, Philipps-University Marburg, Marburg, Germany; ^2^Department of Educational, Social and Organisational Psychology, School of Psychology, Makerere University, Kampala, Uganda; ^3^German Federal Institute for Occupational Safety and Health (BAuA), Dortmund, Germany; ^4^Department of Psychology, Work and Organisational Psychology, University of Bern, Bern, Switzerland

**Keywords:** autonomy, recovery, strain, mental health, small business owners, entrepreneurship

## Abstract

While research on personality factors and economic success of entrepreneurs has flourished over the years, studies on their specific working conditions and their impact on health and career are surprisingly scarce. This study used a qualitative approach to comprehensively mirror the working situation of German small business owners. To reflect the broadness of this employment type and avoid sampling bias, we applied a quota sampling strategy based on a preliminary typology of solo self-employed respondents we derived from a large quantitative survey. We investigated 29 small business owners who reported, for example, on health complaints, recovery opportunities, and obstacles and resources while running their businesses. Thematic analysis was employed to develop a specific frame model for small business owners based on established work-related stress theories which allowed us to derive concrete hypotheses for further quantitative research. The main results emphasized the meaning of active actions and the workers’ own responsibility for creating working conditions and enabling autonomy. Besides personal preferences regarding the chosen career path, marketability, flexibility, and social networks played a role and explained health and career issues. When it came to practical implications, voluntariness played an essential role for selecting this specific career path. Those being pushed into self-employment as their only viable job opportunity should receive particular support through career counseling to sustain their health.

## Introduction

Much research has been done on the economic effects of self-employment, environmental conditions for entrepreneurial success, and if the attributes of the person themselves fit into this career path. Successfully running a business is contingent on the health of the entrepreneur. Small business owners (being solo self-employed without personnel) face financial uncertainties, a high workload, long working hours, and are often unable to call in sick. The financial uncertainties and economic insecurity most small business owners face daily recently became apparent with the emergence, spread, and impact of the COVID-19 crisis. This led to the creation of policies to offer specific support for those who were solo self-employed. For example, under the label of “We won’t leave anybody alone,” the German government has agreed on an emergency aid package worth 50 billion euros to support micro enterprises, freelancers and one-person businesses (Presse- und Informationsamt der Bundesregierung, 2020). Hence, our study aimed to explore the working situation, assuming that resources (e.g., autonomy), strain (e.g., dependency on contracts with clients) and health and career outcomes of small business owners come at the right time.

Small business ownership contributes to the creation of workplaces and new products, and is therefore important for a country’s economic development (Carree et al., 2002; Thurik and Wennekers, 2004). Recently, there has been renewed interest in entrepreneurship (Nabi and Holden, 2008) partly in response to the economic and unemployment crises (Urbanos-Garrido and Lopez-Valcarcel, 2015; Vogel, 2015; Santos et al., 2017). An estimated 10% of the working population in Germany is self-employed or owns a small business (Carter, 2020). While this group is comparably large, research so far has mainly neglected the study of the specific situation of small business owners. In this paper, we equate small business owners to solo self-employed people. Solo self-employment can be defined as operating a business and having the sole responsibility for one’s economic success without employing others for technical or professional support. Solo self-employed people have to be differentiated from employer entrepreneurs who are self-employed as well but utilize personnel (Schummer et al., 2019). Notably, to be counted as solo self-employed for tax purposes in Germany, helping family members as well as other services (e.g., cleaning staff) are not counted (Brenke, 2013).

With our study, we would like to shed light on the working conditions of the solo self-employed as it might play an important role for their well-being and mental health. Although extant research shows that the self-employed tend to report high levels of happiness and well-being (Binder and Coad, 2013; Schneck, 2014; Markussen et al., 2018), self-employment involves numerous challenges such as risk and long work hours that could threaten their mental health (Baron et al., 2016). These working conditions depend on the market situation and are determined by individual differences regarding the motives for their selected career path. Despite increased research focusing on entrepreneurs’ psychological well-being (Ryff, 2019), there is still a paucity of studies exploring the demands and resources arising from solo self-employment. However, demand and resources can heighten or buffer against stress from work. Solo self-employment can be more challenging and demanding than other forms of self-employment, yet small business owners can also enjoy independence and autonomy as they work alone. In contrast, this can also be the cause for some mental health challenges, such as loneliness and lack of social or emotional support. Therefore, we conducted an interview study that aimed to develop a work psychological stress model for solo self-employed individuals based on their lived experiences.

In the following chapters, we will derive our three main research questions (RQs) by first referring to the well-being of small business owners, introducing the interplay of stressors and resources and its impact on mental health for small business owners, and we finally summarize relevant knowledge on the role of personality in well-being. Hence, we look at both work-related and personality factors and how they shape well-being.

### Well-Being and Mental Health of Small Business Owners

Recognizing the value of well-being to humans functioning at work, Article 24 of the Universal Declaration of Human Rights focuses explicitly on recovery and sustaining physical and mental health. More specifically, it declares that people should have the right “to rest and leisure, including reasonable limitation of working hours and periodic holidays with pay.” Across most countries, restrictions on working hours, quantity of vacation days, as well as duration and frequencies of breaks during working time are protected by legislation and included in employees’ working contracts – at least for employees who are regularly employed.

Work is one of the most important aspects of human life, and it is therefore important to psychological development and function (Blustein, 2008). Nevertheless, it also has the potential to thwart psychological function in the case of undesirable experiences in one’s work life. Work is essential for gratification of the basic psychological needs of autonomy, competence, and relatedness (Van den Broeck et al., 2016) which in turn foster psychological growth and well-being (Ryan and Deci, 2001; Deci and Ryan, 2008; Van den Broeck et al., 2016). In terms of self-employment, existing research indicates that entrepreneurs tend to be happier and report high levels of psychological well-being (Binder and Coad, 2013; Baluku et al., 2018a; Shir et al., 2018; Nikolaev et al., 2019). In contrast, individuals tend to experience serious mental health challenges such as low self-esteem, substance abuse, and severe mental health concerns when they are out of work (Blustein, 2008; Otto and Dalbert, 2013). Such experiences are not uncommon in self-employment, given that an entrepreneur has to work long hours while undertaking the complex process of starting a venture and going against competition in the business space (Baron et al., 2016).

An important outcome of work, which is one of the major attractions of self-employment, is autonomy (van Gelderen and Jansen, 2006; Schneck, 2014; Jubari et al., 2017; Baluku et al., 2019). This is one of the essential goals that people seek to achieve in their workplaces. However, this outcome is generally threatened by digitalization. There is an increased risk of constant accessibility through digital apparatuses, including the internet and smartphones. This makes it more difficult for employers to separate work from the family domain in order for employees who prefer privacy to recover (Derks et al., 2016). Overall, people who are self-employed, working on-demand, in portfolio careers, or the “gig-economy,” often experience little protection, and may even violate their own rights regarding work-family life balance in order to maintain their jobs, customers, or overall employability (Fleming, 2017). Similarly, there is an increasing risk of abuse of independence or autonomy at work among the self-employed. Being one’s own boss, coupled with high demands from customers, increases the temptation to work longer, often on weekends and holidays, increasing the risk of exhaustion, diminished relatedness, and stress.

In some forms of self-employment (e.g., solo self-employment), individuals willingly work extra hours with or without being conscious of the implications for their well-being. This facilitates the experience of negative emotions which include fear, strain, and stress. Although these effects may be dependent on regulatory coping behaviors (Patzelt and Shepherd, 2011). The knowledge of health and well-being of small business owners is an important topic to explore. Accordingly, our first research question is as follows:

RQ1: How do small business owners perceive their health status? Do they have sufficient opportunities and time to recover from work? What happens to them in case of sickness?

### The Interplay of Stressors and Resources for Well-Being

Despite its positive psychosocial functions, work can be an important source of stress. The term stress refers to a subjectively unpleasant state of strain arising from the fear of being unable to cope with an aversive situation (Zapf and Semmer, 2004). Lazarus and Folkman’s structural model of appraisal (1984) is one of the most common models in stress theory. According to the authors, cognitive processes steadily evaluate the current situation regarding its meaningfulness for one’s well-being. They differentiate between three kinds of appraisals: the primary appraisal, the secondary appraisal, and the reappraisal. During a person’s primary appraisal, he or she evaluates whether the current situation is important for his or her well-being. The situation can be interpreted as positive, irrelevant, or dangerous. In both positive and irrelevant situations, there is no need for action. If a situation is interpreted as dangerous, actions need to be taken to sustain or retrieve one’s well-being. In this case, available resources are analyzed in the stage of the secondary appraisal.

Resources can be material, social, physical, or psychological. In other words, resources are factors that are directly or indirectly of value for survival or that lead to the attainment of such value (Hobfoll, 1998). If the person has sufficient resources to cope with the situation, it is perceived as a challenge from which the person can learn or profit in another way (cf. LePine et al., 2005; Widmer et al., 2012). If the person, however, does not have sufficient resources he or she perceives stress, which may result in negative consequences for his or her well-being. This stressful situation can now either be coped with using a problem-focused approach, meaning to act and thereby to change the situation itself, or an emotion-focused one, meaning to change the relation to the situation or to adjust to it (e.g., Lazarus, 1999; Semmer, 2003). The process ends with the reappraisal, which monitors the situation repeatedly and takes care of necessary behavioral adjustments to changing situational characteristics.

The emotions people experience in those situations depend on how they perceive their ability for problem-focused or emotion-focused coping and what is an appropriate response to the situation (cf. Zapf and Semmer, 2004). Thus, personal resources such as self-esteem can buffer negative consequences of social-evaluative threats (Dunkel Schetter and Dolbier, 2011; e.g., by facilitating faster habituation; Elfering and Grebner, 2012). Nevertheless, under conditions of limited stress exposure and successful recovery (cf. Geurts and Sonnentag, 2006; Geurts, 2014), stress exposure itself could have a strengthening effect on the individual (toughness; Dienstbier, 1989; Seery et al., 2010; Ganster and Rosen, 2013). However, if the exposure is not transient, chronic stressors could reduce resource capacity and impair coping (e.g., Elfering et al., 2005) – increasing the vulnerability to stress. Thus, beyond the source of stress, the possibilities to recover and the person’s ability to recover are also relevant.

Previous knowledge of work psychology, which mainly comes from studies with paid employees, is the basis utilized when it comes to understanding the health and performance of self-employed people. We assume that stressors and resources in solo self-employment differ as indicated, for example, by the fact that people work solo without any co-workers, superiors, or subordinates. Taking these considerations as a basis for relevant concepts and processes with this research, we aimed at developing a specific work psychological model for small business owners as suggested by our second research question.

RQ2: Which stressors shape the working situation of small business owners? What resources do they experience in their work? How do both types of job characteristics – i.e., stressors and resources – interplay when explaining well-being?

### The Role of Personality for Well-Being of Small Business Owners

Small business owners are more strongly responsible for creating their working conditions (on their own) favorably as compared to employed people or employer entrepreneurs (i.e., self-employed people with personnel; Schummer et al., 2019). Also, when it comes to health, stress and strain have individual differences. These emerge and can be traced back to several relevant psychological concepts. For this paper, we limited our review to three concepts that are important to our findings: including personality, psychological resources (psychological capital) specifically self-efficacy, and motives or goals.

Person-environment fit theories have been applied to understand why some people choose, persist, and succeed in self-employment or entrepreneurial careers. Focusing on the theory of vocational personalities and work environments (Lasser, 1974), each of the six personalities represents a set of interests, preferred activities, beliefs, abilities, values, and characteristics (Nauta, 2010) that must be congruent to the environment or the characteristics and realities of a given profession or work. In line with our study focus on “demands and resources in solo self-employment,” enterprising individuals tend to be adventurous, acquisitive, ambitious, energetic, optimistic, confident, and sociable (Spokane and Cruza-Guet, 2005). More recent research has advocated to focus on less stable personality traits or constructs such as risk taking ability or risk tolerance, need for achievement, personal initiative, proactivity, and flexibility, respectively (Rauch and Frese, 2007; Obschonka and Stuetzer, 2017; Baluku et al., 2018c). On the one hand, all these qualities may be important resources in different activities or stages of the business process. On the other hand, a lack of these qualities may represent person-environment incongruence which increases the likelihood of strain, stress, and consequently lowered well-being and work satisfaction.

The entrepreneurial process is complex and each stage of the process comprises of challenging tasks that are potential triggers of stress (Baron et al., 2016). The process is even more demanding for the solo self-employed who must perform all business tasks by themselves. Consequently, a significant amount of psychological resources is required to manage and cope with such work pressures. These resources are constituted in the construct of psychological capital (Goldsmith et al., 1997; Luthans et al., 2004; Luthans and Youssef-Morgan, 2017), which has been found to significantly predict low levels of stress among entrepreneurs (Baron et al., 2016). However, it is not known yet whether this generally applies to all self-employed people, or even small business owners such as the solo self-employed. Based on the positive psychology literature, psychological capital comprises four resources, including self-efficacy (confidence), optimism, hope, and resilience (Luthans et al., 2004, 2007; Luthans and Youssef-Morgan, 2017). It has been suggested that when combined, these resources make a stronger contribution to business success and persistence than tangible, human, and social capitals (Luthans et al., 2004; Baluku et al., 2016, 2018b). Accordingly, psychological capital provides the mental hardiness needed to cope with the work demands involved in self-employment (Baron et al., 2016). Each of the resources involved play different yet complementary roles. Baron et al. (2016) explain, for example, that self-efficacy helps to reduce experienced stress while the positive expectations involved in optimism helps mitigate the stress. Hope is useful in developing multiple pathways to overcome the work challenges and resilience enables individuals to persist in overcoming challenges.

Individuals also differ in their motives and goals for engaging in entrepreneurial activities or small businesses. To some, it is income or the opportunity to create wealth, while to others, it is about the freedom of being one’s own boss in contributing to or bringing about a change in society. To others, it is just an employment option that is better than being unemployed. However, the self-determination theory provides an educated framework for understanding human motivations and goals for engaging in different behaviors including work. From this perspective, it is logical to assert that the self-employed seek more than just monetary outcomes (Hamilton, 2000). Rather, and in line with the realities of protean careers (Hall, 2002; Briscoe and Hall, 2006), individuals seek to gratify psychological needs including autonomy, competence, and relatedness (Deci and Ryan, 2000; Deci et al., 2001; Gagné and Deci, 2005). Particularly, autonomy seems to be what most people strive for in the workplace as it facilitates the achievement of organizational goals and personal agendas such as well-being (Hodson, 1991; Gagné and Bhave, 2011; Otto et al., 2013). When psychological needs are satisfied, it results into greater self-motivation, engagement, and volition and consequently creativity, superior performance, and persistence (Deci and Ryan, 2000; Ryan and Deci, 2000; Gagné and Deci, 2005). Hence, gratification of psychological needs can represent further psychological resources for work. However, if self-employment is not facilitating the gratification of these needs, it can result in the experiencing of psychological strain and a lowered well-being. Relatedly, individuals also differ in which pursuit they have in terms of choosing between income or wealth. Attaining financial security to meet familial and other financial needs could boost job resources among the self-employed.

In conclusion, individual differences in personality, psychological resources, goals and motives exist which might have a direct impact on well-being or an indirect impact via evaluating stressors and resources which affect health in turn. Accordingly, we were interested in the role of individual differences for explaining well-being for the solo self-employed leading to our third and last research question.

RQ3: What were the motives for becoming (solo) self-employed? Were the small business owners attracted (push) by this type of employment or did not have a different choice (pull)? Does the product or service as well as the conditions in the market play(ed) a role?

## Methodology

We regard the group of solo self-employed as experts in their field and aimed to conduct expert interviews (Bogner et al., 2009). According to Meuser and Nagel (2009), “an individual is addressed as an expert because the researcher assumes (…) that she or he has knowledge (…) which is not accessible to anybody (…). It is this advantage of knowledge which the expert interview is designed to discover, and it is an exclusive realm of knowledge which is highly potential because and in as far as it is linked with the power of defining the situation” (p. 18).

### Sampling Criteria and Sampling Process

As discussed by Robinson (2014), sampling is central for best practice in qualitative research and for its impact and trustworthiness, which calls for a clear (1) definition of a *sample universe* through inclusion and exclusion criteria for potential interviewees; a (2) decision upon a *sample size* by balancing out research-based interests and practical concerns; a (3) well-reasoned selection of a *sampling strategy* as well as (4) *sample sourcing* containing issues of advertising, incentivizing, avoidance of bias, and ethical concerns.

The *sample universe* is set by the definition of solo self-employment indicating that potential participants should run a business and have the sole responsibility for their economic success without employing others for technical or professional support except helping family members (Brenke, 2013).

As solo self-employed people can work in nearly all professional fields, we developed a typology to assess all or at least most of the relevant types of small business owners prior to our interview study (Kottwitz et al., 2019a). This typology was derived from data of a large quantitative survey which also included (solo) self-employed individuals. That way, we tried to avoid bias in *sample sourcing* by making sure that we do not neglect specific types or overestimate them (cf. Robinson, 2014). Specifically, the solo self-employed people to be approached to participate in our expert interview study (Bogner et al., 2009) should mirror the broad range—with respect to demographic factors, prestige, qualification, and job insecurity—within the chosen profession.

We used the data from the Federal Institute for Vocational Education (BIBB)/German Federal Institute for Occupational Safety and Health (BAuA)’s employment survey of the working population on qualification and working conditions in Germany as collected in the year 2012 to conduct cluster analysis and to derive a typology of small business owners. About every 5 years, the BIBB and the BAuA jointly conduct this representative survey. Data were available for 20,036 volunteers who were above the age of 15 years and worked at least 10 h per week (Rohrbach-Schmidt and Hall, 2013). Overall, data of 883 solo self-employed people were available, and their clusters were analyzed and used for the following cluster analysis.

*Age* and *gender* play important roles when it comes to: (solo) self-employment (e.g., Smith and Tolbert, 2018). The mean *age* of the solo self-employed in the sample was 49.68 years (*SD* = 11.69), and there were 501 women and 382 men. Besides these two demographic factors, we took the *professional qualification level* into account as it is relevant for our typology. Moreover, we considered the *prestige* of the occupational activities of the small businesses by using the magnitude prestige scale (MPS, Wegener, 1982, 1983) which evaluates the societal reputation of a profession. In the group of solo self-employed of the BIBB/BAuA employment survey, the lowest value was given for agriculture workers in men (30.10) and cleaners in women (32.20) and the highest for doctors or pharmacists (191.30). Finally, there might be important differences regarding voluntariness of, and hence commitment to, the self-employed role (Baluku et al., 2018a,b). As there were no data available concerning the willingness to stay in solo self-employment, we used the *unemployment quote by gender* as a proxy to have an indicator of a range of difficult economic situations and thus higher or lower employment stability. The unemployment quote ranged from the field of “theology and community work,” with 0.10% for women and 0.30% for men, respectively, to 42.50% for women in the field of textile work; the mean unemployment quote was 10.31% (*SD* = 8.26%).

Considering the five aforementioned criteria, we derived the following 11 types of solo self-employed people that were relevant to interview: (1) Female with uncertain status and comparably low qualification level (*n* = 40, typical professions: cosmetician, assistant in health care, textile processor), (2) Male with uncertain status (*n* = 85; typical professions: insurance agents, roofer, building technician), (3) Low qualified young female (*n* = 33; typical professions: nanny, childminder, learning supervisor), (4) Low qualified young male (*n* = 36; typical professions: carpenter, glazier, photo technician), (5) Young high potential male (*n* = 17; typical professions: journalist, software engineer), (6) Older solo self-employed with low status but secure employment (*n* = 174; typical professions: various with low unemployment risk), (7) Low status but secure (*n* = 115; typical professions: various with low unemployment risk), (8) High qualified and secure status (*n* = 120; typical professions: business consultants), (9) Older highly qualified and secure status (*n* = 195; typical professions: business consultants), (10) Highly prestigious female professionals (*n* = 42; typical professions: psychotherapists, dentists, attorneys), and finally (11) Highly prestigious male professionals (*n* = 26; typical professions: dentists, physicians, legal advisers).

As for the *sample size*, we aimed to interview between 25 and 30 small business owners. Regarding sampling strategy, we chose a *quota sampling strategy* (Robinson, 2014) based on the percentage of solo self-employed in each category of the typology (as was indicated in brackets above). Accordingly, we planned to approach and interview *n* = 3 small business owners of category (1), *n* = 4 of category (2), *n* = 1 of category (3), *n* = 2 of category (4), *n* = 1 of category (5), *n* = 7 of category (6), *n* = 4 of category (7), *n* = 4 of category (8), *n* = 6 of category (9), *n* = 2 of category (10), and *n* = 1 of category (11). Except for category (1) where we interviewed only *n* = 2 (instead of 3) people, and categories (6) and (7) where due to saturation in the interviews we only questioned *n* = 4 (not *n* = 7) and *n* = 2 (not *n* = 4) solo self-employed, respectively. Our selection of participants was similar to the one required. While this typology itself should be treated with caution (as relevant indicators as voluntariness might not be perfectly reflected) it was justified for us to use it as a guideline to select participants and to avoid bias in sampling (Robinson, 2014). We created flyers and websites for advertising purposes, received ethical approval and used networks of self-employed people (e.g., unions) to gain access to our experts.

### Sample Description

By use of quota sampling and based on the typology explained above, we contacted 40 solo self-employed and asked for their willingness to be interviewed. Of these, 29 agreed to participate, leading to a response rate of 72.5%.

More precisely, we interviewed 11 women and 18 men, of which 27 had a German citizenship. Their age varied between 25 and 84 years old (*M* = 52.86; *SD* = 13.31). More than two-thirds (*n* = 17) of our sample had attended a university or technical college. Eleven small business owners had to deal with unemployment experiences in their past while the same number had received financial support to start their businesses. On average, the interviewees had been solo self-employed for 215.76 months (*SD* = 174.13).

When it comes to the sectors represented, the majority (20 out of the 29) of interviewees owned their small businesses within the service sector. Other sectors included the fields of adult education, personnel and organizational development, software engineering, and health professions. The remaining participants worked in the fields of commerce, handicrafts, industry, and arts. Twenty-one interviewees run a business in an occupational activity which was completely similar to their study or vocational training, while for another three their study and final careers were slightly less related. For the remaining five small business owners, their practiced occupational activity had nothing to do with their prior qualification.

### Interviews and Data Analyses Procedure

All participants were interviewed face-to-face by six trained interviewers. Each interview lasted, on average, 58 min and 50 s (*SD* = 19 min and 53 s). The participants gave permission for the recording of the interviews and this was accomplished using digital recording equipment. Using partially standardized interviews, the interviewees were asked questions about eight major topics. In addition to general information about their occupational activity, these concerned (a) their motives as well as goals and their achievement, (b) perceived advantages and disadvantages of solo self-employment, (c) their adaptability and how to deal with change, (d) working time and the balancing of work and private life, (e) social structures, (f) burdens and resources, and (g) their perceived health, success, and performance. In addition, (h) they were interviewed about their wishes for occupational safety and health which might be particularly relevant for the future design of solo self-employment (see practical implications).

We explored our research questions by use of *thematic analysis*; this is a method involving searching across a data set to find repeated patterns of meaning by constantly moving back and forward between the entire data set, the coded extracts of data as well as the analysis of the data produced (see, Braun and Clarke, 2006). As suggested by Mayring (2015), the audio recordings of the interviews were professionally transcribed as a first step. During the process of data analysis, the transcripts were regularly checked back against the original audio recordings for accuracy and refinement.

As to the level of analysis, we used a *semantic approach* in which themes are identified within the explicit or surface meanings of the data without looking for anything beyond what our experts had said. Regarding the type of analysis, we aimed at providing a *rich thematic description* of the interviews allowing a reader to recognize the important themes limiting potential depth and complexity which according to Braun and Clarke (2006) “might be a particularly useful method when (…) investigating an under-researched area” (p. 83).

Overall, our analysis was guided by established stress-theoretical models (deductive approach) but we also looked for data-driven themes (inductive approach). In doing that, our data analysis started with identifying the key phrases from an arbitrarily chosen interview. Considering theory-driven scientific knowledge, but further following the technique of inductive category development, preliminary categories were formed when working through this first interview. They were then revised and refined in the process of coding the remaining interviews.

Finally, for reasons of quality control and to optimize our findings, we applied the method of communicative validation (Kvale, 1995). Specifically, after the interviews were analyzed, with themes identified and a preliminary model developed, we invited our participants to an expert meeting to discuss and refine our model and hence guarantee its validity.

## Results

### Well-Being and Strain

We aimed to develop a work-psychological stress model specific for solo self-employed people containing but also going beyond the conceptualizations of prior stress theories. Accordingly, our first research question was focused on the well-being and strain situation of solo self-employed people. In line with the World Health Organization (WHO), we define health as a “state of complete physical, mental and social well-being and not merely the absence of disease or infirmity.” To reflect this point broadly, we first asked our interviewees about their general state of health on a quantitative scale. We then asked about their perceived possibility to calling in sick and recovering in case of illness. This was a concrete outcome of health closely linked to their business situation.

On a five-staged measure, five of the 29 interviewees reported that their health status is “very good,” 18 said it was somewhat “good,” three were “undecided,” two answered about having a “somewhat bad,” and one solo self-employed even indicated a “very bad health” status.

Our model should ultimately explain how to maintain health for the solo self-employed. One of the main important points to “repair” or sustain well-being is the opportunity to recover. Recovery can be seen as a central mechanism that translates the characteristics of the work into possible consequences. In this respect, we regard recovery as proximal to the maintenance of health. Psychological research has yielded a broad consensus that adequate recovery is needed (Zijlstra and Sonnentag, 2006) to sustain one’s health and productivity (Geurts and Sonnentag, 2006).

As recovery seems to be the key to well-being (Geurts, 2014), we asked small business owners in our sample about what happens in case of illness and if they have enough time to recover. While some of the solo self-employed affirmed that they are able to recover, others denied it or admitted that it depends on the circumstance. Hence, we derived three main categories with more detailed sub-categories. The categories, sub-categories, and sample phrases as reported by the interviewees are summarized in Table 1.

**TABLE 1:** Solo self-employment and time for recovery in case of illness.

**Category**	**Sub-category**	**Example**
(1) Yes, time to recover	(a) Unconditional agreement	“That’s a mental question. I guess it’s ultimately absolutely brainwork to do that. There’s a little trouble when I get sick, but then I throw a switch and crawl into bed without having a bad conscience. Sometimes I am happy to be able to take time off and to withdraw from somewhere and wait until I get fit again. This is how I do it.” (*male, 57 years, 32 years solo self-employed*)
	(b) The restriction that someone is rarely ill	“Illness conflicts with my self-employment and I can take time for recovery. Astonishingly enough, I have worked in an organization for almost 20 years before I started my own business. In these 20 years and earlier, I guess, I was more often ill than in self-employment and that’s what I find interesting. Additionally, in these 20 years, I very rarely said that I was not coming due to illness. But, I agree, I would do it. By the way, it would not work at all – Once I’m ill I have no chance of doing what we are doing. I might still be able to work representationally, but not in that field. We better cancel and my decision will be accepted.” (*male, 59 years, 20 years solo self-employed*)
	(c) Yes, but it was not like that in the past	“When I started teaching, I always thought that if I’m not there, the whole chain would break down. Therefore, I also taught sick. I don’t do that anymore, I really take time to cure myself, because it’s no good for me or anybody else.” (*female, 27 years, 7 years solo self-employed*)
(2) No time to recover	(a) No, working despite illness	“No!” (*male, 40 years, 8 years solo self-employed*)
	(b) No, someone is rarely ill	“I have not been ill for 17 years now. If I would get ill, I would really be in a dilemma. That really wouldn’t work. Maybe for 1 week, 2 weeks would already be a catastrophe. If I would be really ill, I would be broke immediately or even dependent on income support. From 1 day to the other. Dead tomorrow.” (*male, 53 years, 17 years solo self-employed*)
	(c) No, but working more carefully with personal resources	“I have been ‘ill’ for 1.5 years now, ‘ill’ with quotation marks, and therefore I didn’t accept too many orders, only standard seminars which I already knew about.” (*female, 32 years, 4 years solo self-employed*)
(3) Depending on the circumstances	(a) Duration of illness	“Well, I can manage my time. However, to actually have enough time to cure myself, that’s another question.” (*male, 84 years, 51 years solo self-employed*)
	(b) Type of illness	“If I see no other way out, yes, of course. However, in case of a non-serious illness, I usually go to work sick. If I have a cold or flu unless my head is really closed now, I can definitely stay at home for 1 day and have to cancel all patient appointments.” (*male, 54 years, 11 years solo self-employed*)
	(c) Business situation	“Depending on whether there are any important deadlines, then definitely not. In general, however, you can arrange things so that there is enough time. It’ll be fine.” (*male, 47 years, 1,5 years solo self-employed*)

For those agreeing that they *have time to recover*, the solo self-employed respondents gave unconditional agreement (1a), approval with the restriction to being rarely ill (1b) or approval but granting that it was not like that in the past (1c). Hence, it seems that for some people, there has been a development which might result either from a general demarcation or because of professional success. In cases where it was stated there would be *no time to recover*, the interviewees either worked despite being ill (2a), quoted that they would be rarely ill at all (2b), or expressed that they would work more carefully and conserved personal resources (2c). Moreover, if people stated that it *depended on the circumstances* it was the case that either the duration of the illness (3a), the type of the illness (3b), or the specific business situation, i.e., the order situation, determined whether recovering from diseases would be possible or not.

Regarding the first research question, it can be summarized that it is necessary to more strongly explore the health situation of the solo self-employed. The interviews underlined that even if there is a need for recovery, the small business owners do not always have or take the opportunity to detach themselves from their businesses. Some even reported that they worked even if they were actually too sick to do so. This phenomenon is known from research with those who are paid employed and is known as presenteeism. This refers to the act of working while being ill (Johns, 2010) and was shown to have negative effects on work ability (Gustafsson and Marklund, 2011) and health. This also led to an increased risk of emotional exhaustion (Taloyan et al., 2012).

### Job Characteristics in Solo Self-Employment

To answer our second research question, i.e., analyzing the specific positive and negative aspects of the work situation, we explored the job characteristics of solo self-employed people. In contrast to other forms of employment (i.e., self-employed with personnel, employed in a company/public institution), the working situation of solo self-employed people is reflected by their sole responsibility for each and every part of their job. Hence, they can be the driver or in charge of healthy workplaces for themselves. They have to build social networks to get support because they have no co-workers, and they have sole autonomy which might be both a blessing and a curse.

**FIGURE 1:**
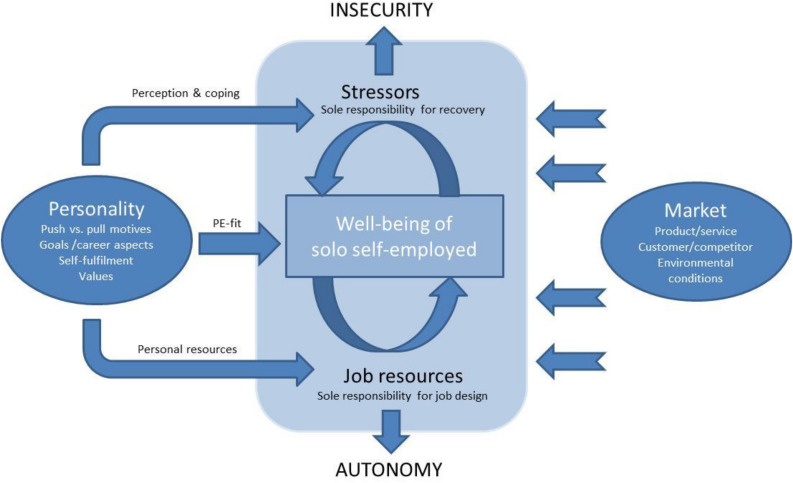
A work-psychological stress model for small business owners.

Autonomy in solo self-employment is closely linked to the demands of sole responsibility—indicating that stressors and resources seem to merge. Moreover, autonomy is created not only by self-employed work within a specific market and product context itself, but also sets its boundary conditions by its market rules, customer needs, or supplier conditions. Moreover, personality factors play a key role in explaining whether people choose employment with such high levels of responsibility and autonomy and whether they are satisfied and committed to it. This complex model is illustrated in Figure 1.

In the following chapter, we will describe parts of the model by introducing (1) work-related demands shaping the working situation, as well as (2) work-related resources helping to deal with it, considering how they are related to: (3) strain, health and recovery as a consequence of the stressor−strain relationship.

#### Demands Arising in Solo Self-Employment

According to the job demands-resources model (JD-R) (Demerouti et al., 2001), demands are related to strain and described as “physical, psychological, social, or organizational aspects of the job that require sustained physical and/or psychological (cognitive and emotional) effort or skills and are therefore associated with certain physiological and/or psychological costs” (Bakker et al., 2006, p. 312). In the interviews, the sole responsibility for all working aspects was seen as the key point as shown by answers to the question “what, broadly speaking, usually are the things which are demanding or maybe even burdening in the job”? Overall, we crystalised five different categories of demands (or stressors), with each further containing various sub-categories. A detailed description of the categories, sub-categories, and sample phrases can be found in Table 2.

**TABLE 2:** Demands arising from solo self-employment.

**Category**	**Sub-category**	**Example**
(1) Task responsibility	(a) Sole responsibility	“One disadvantage is that you are on your own…and solely responsible for everything you do, have to do or want to do.” *(female, 55 years, 10 years solo self-employed)*
	(b) Tasks outside occupational core tasks (misfit to occupational role)	“I would like to have someone to delegate organizational stuff to in my team. However, for me this would only be economically viable if I would work with several colleagues in a practice. In view of organizational effort, writing reports, telephone service, consultation hours and so forth…” (*female, 43 years, 6 years solo self-employed*)
	(c) Unnecessary tasks (senseless, dictated from outside)	“Things which do not result from workflow or a project, but which one actually has to do – i.e., posting things more frequently in social media or writing an article - not because it’s necessary or it has been on my mind for some time now, but because it has to be done again. Thus, actually externally controlled and required by the outside world.” (*male, 61 years, 24 years solo self-employed*)
(2) Temporal responsibility	(a) Time and performance pressure	“The other point includes rather an over-load in projects in which you have to provide an intensive service within a short period of time. These are real stress factors; I would say that this is the worst experience you can make.” (*male, 47 years, 12 years solo self-employed*)
	(b) Flexibility overload	“As concerns flexibility, it means a shortcoming to me, if you can’t limit yourself just a bit because at the end you say: ‘Let’s also do this and that.’ And, as a result, you easily have a 55−65 h week and you are facing administrative matters and accounting problems.” (*male, 40 years, 8 years solo self-employed*)
	(c) Lack of time for preparatory work and training	“Sometimes a little bit more time, a stress factor aroused by the fact that I have to manage an essential part of the income and consequently only have little time to familiarize myself with training. Reading, for example, – I always have a number of great books but I don’t get around to reading them.” (*female, 43 years, 6 years solo self-employed*)
(3) Responsibility for personal success (product responsibility)	(a) Task related uncertainties	“At one point you realize a little bit more surprisingly that the crux of this matter is the handling of not-knowing. Things you don’t know about will hit you. These are risks…the risk factor and your own dealings with it.” (*male, 59 years, 20 years solo self-employed*)
	(b) Conflicts of values	“I experienced it twice, that people during a seminar are not receptive to argumentations at all. Nowadays, the lack of receptiveness almost seems to be normal when you try to discuss with some Pegida* people or others, i.e., famous alternative facts you cannot reach anymore. I am just a qualified natural scientist. I like working with facts and logic. But, however, you cannot reach some people. I have a problem with this kind of people attending a course, which was paid for them. These are all things, which really weight on me.” (*male, 59 years, 14 years solo self-employed*)
	(c) Handling of difficult customers (failures, critic)	“General conflicts with a customer, which are very rare, but, however, weight on me. If, for example, strong mistrust or criticism arises.” (*male, 57 years, 32 years solo self-employed*)
	(d) Imbalance of effort and reward	“I think time expenditure is onerous as I work very long hours. Compared to the low income - I think you could be paid a lot better considering a 60 h week.” (*male, 40 years, 8 years solo self-employed*)
(4) Responsibility for economic success	(a) Self-marketing	“That’s what marketing is all about: acquisition, doing things up to the point I am facing a human, interested person with the ability to communicate – then, acquisition, writing offers and developing concepts don’t cause me any problem. Compared to initiations of business connections and everything that might happen in an open space.” (*male, 61 years, 24 years solo self-employed*)
	(b) Financial uncertainty (cost coverage)	“Direct disadvantages. Yes, sure, self-employment always means a financial game. You never know what a month will look like: will there be any incoming orders. That is always a bit of a problem. Sure, expenditures are rising continuously each month and they sometimes don’t go with the expenses, therefore you always have to vary accordingly, thinking of how you could balance expenditure again.’ (*male, 58 years, 2 years solo self-employed*)
	(c) Future prospects (job insecurity)	“Insecure order situation, noticeable dependence on a relatively few number of clients, a standing still feeling. The feeling of no actual further development. You just have the feeling that it cannot go on like this. In this sector, you somehow come up against limiting factors, payment comes up against limiting factors. You just have the feeling that development potentialities are extremely limited. If then, in addition, you have the feeling of a step backward…At a certain age you don’t have the impression of rising strength.” (*female, 55 years, 25 years solo self-employed*)
	(d) Social security (savings)	“If I don’t work, I don’t have any income. I don’t have paid holidays, no continued payment of wages in case of illness. These are disadvantages, I guess.” (*male, 54 years, 11 years solo self-employed*)
(5) Sole design of interactions in social structures – social problems	(a) Conflicts with colleagues	“Yeah, well, I was also told: ‘What do you actually want? Give it a rest. You only take this place away from others. You don’t need it. What do you actually want?’ That’s really sad and I ask myself: how deep does a doctor has to fall to say, think or feel something like that.” (*female, 57 years, 24 years solo self-employed*)
	(b) Conflicts with external suppliers/workers	“As a self-employed person you are always stressed – especially in the decisive phase when tasks are to be handled and completed. That’s the reason why I sometimes express myself very negatively in some contexts. Why hasn’t this been done? Do I have to say that or give reasons a thousand times? Why has the invoice not been issued correctly? Have a look, if this is about 12 euros now and… More and more prices to make, actually nobody was talking about at all. I’m annoyed about these things, of course.” (*male, 84 years, 51 years solo self-employed*)
	(c) Delegation not possible	“Uncertainty as a whole, planning and everything that goes with it. As an employer, you might more easily say, for whatever reasons: ‘Please, do it; I don’t want to.’ or whatever. They are able to delegate much more. I cannot. Sure, I could instruct myself (laughs). But this is maybe the small advantage for employers.” (*male, 58 years, 2 years solo self-employed*)
	(d) Lack of social exchange	“First of all, there’s primarily nobody there to talk to. I think that this in itself is a burden. And the fact that I am the only person solely responsible for certain things.” (*male, 54 years, 11 years solo self-employed*)

*Pegida, Patriotic Europeans against the Islamization of the Occident: Pegida believes that Germany is being increasingly Islamized and defines itself in opposition to Islamic extremism (Taken from: The End of Tolerance? Anti-Muslim Movement Rattles Germany. Der Spiegel. December 21, 2014).

The categories 1 to 3 refer to aspects that are part of paid employment as well. However, the solo self-employed are special because they are shaped through their obligation to create and execute all work tasks themselves, handle time management, and face all possibilities of success and failure. The first category of *task responsibility* illustrates that the small business owner is in charge of all tasks (no matter whether the task is professionally adequate or goes beyond his or her professional knowledge), i.e., has the sole responsibility (1a). Moreover, beyond such legitimate tasks that come from a different professional field yet, the solo self-employed also reported being in charge of tasks that are either perceived as unreasonable (1b; misaligned with occupational role) or unnecessary (1c; dictated from outside). Both kinds of tasks must be evaluated as illegitimate tasks (Semmer et al., 2015) as they should not be expected from the person and contain an element of a lack of appreciation (Kottwitz et al., 2019b) leading to mental impairment (Semmer et al., 2015).

The second category of *temporal responsibility* summarizes aspects that are concerned with time. As stated by an interviewee, “Yes, the time. I always get the feeling the time is not sufficient; the time simply flies.” Obviously, the burden of time cannot be shared in solo self-employment and so time and performance pressures (2a) occur. The time strain is further aggravated by the fact that time is equitable to money in business. This is comparably less of a concern in paid employment where a contracted working time is guarded by formal occupational safety regulations that define when an employer can expect his or her employee to work. In contrast, solo self-employment contains the danger of completely exhausting any time constraints (2b; overload) and to make a worker concentrate on tasks that are only immediately relevant for adding financial value (2c; no time for preparatory work and training). Additionally, there is knowledge of changing working strategies to maintain performance in the face of stress within the role of paid employment. For example, this can be seen by the abandonment of actions which are perceived to be of a low priority (e.g., searching for feedback, servicing; see Zapf and Semmer, 2004). This tendency might increase for the solo self-employed who have their economic success in mind, eventually leading to a dangerous balance between health and performance (e.g., McDowell et al., 2019).

The *responsibility for personal success* makes up the third category and is unique to solo self-employed people. Such success is difficult to achieve if demands remain obscure during order fulfilment (3a; task-related uncertainties) or contradict with their own personal values (3b; conflicts of values). Both aspects are well-known as work stressors within the concept of the role stress theory (Kahn and Byosiere, 1992). Feedback regarding work performed in solo self-employment can be given from customers or result from fulfilling the task itself. As customers are classified as a main source of appreciation (Jacobshagen and Semmer, 2009), a difficult customer relationship (3c; handling of difficult customers) might shatter the worker’s self-esteem and cause strain (Semmer et al., 2007). It can also be a burden if the effort put into the job is disproportionate to the gained reward (3d; imbalance) both materially or immaterially in the form of appreciation or contract security. It has been demonstrated that an effort-reward imbalance (Siegrist, 2000) may cause emotional distress, potentially leading to the development of physical (e.g., cardiovascular) and mental (e.g., depression) diseases (Van Vegchel et al., 2005).

Next, the fourth category is concerned with the *responsibility for economic success* and relates to the design of the conditions which enable success. The solo self-employed oversee creating and securing economic success to enable their (and perhaps even their family’s) living. Self-marketing (4a), and financial uncertainty (4b) refer to the recent income generation and cost recovery and reflect the current situation the person evaluates. In contrast to that job insecurity (4c; future prospects), and savings (4d; social security) are evaluations concerning one’s future and hence forward-looking.

Finally, the *sole design of the interactions in social structures* goes hand in hand with social problems and conflicts. Interestingly, two-thirds of the interviewees reported making attempts for social involvement in the case of co-working (Spinuzzi, 2012). Obviously, solo self-employed individuals move around in social makeups which partially differ to those of the paid employed, as their interactions are primarily determined by suppliers, customers, and clients. Social problems can emerge at various interfaces with colleagues (5a) or external suppliers (5b) but can also be further caused by the lack of social support. Based on Fisher’s definition (1985, p. 40), “social support is conceptualized as the number and quality of friendships or caring relationships which provide either emotional reassurance, needed information, or instrumental aid in dealing with stressful situations,” and can broadly be differentiated into instrumental and emotional support (e.g., McGuire, 2007). In line with this differentiation, the interviewees complained about not having anyone to delegate tasks to: (5c) as well as a lack of social exchange (5d).

#### Resources Provided by Solo Self-Employment

Following the JD-R model (Demerouti et al., 2001), job resources are related to motivation and defined as “those physical, psychological, social, or organizational aspects of the job that are either/or functional in achieving work goals, reduce job demands and the associated physiological and psychological cost and stimulate personal growth, learning, and development” (Bakker et al., 2006, p. 312). Hence, beyond the demanding aspects of their jobs, the interviewees were questioned about relieving work-related factors, i.e., aspects that are beneficial and aspects that decrease the workload. Table 3 provides the categories and sub-categories of the resources described by the interviewees, with additional sample phrases for each sub-category.

**TABLE 3:** Resources provided by solo self-employment.

**Category**	**Sub-category**	**Example**
(1) Autonomy	(a) Product/customer decision	“Yes, of course, to be able to say no. I think that’s the main point. Relief, yes. Being able to say no and being free to choose for me always means relief.” (*female, 27 years, 7 years solo self-employed*)
	(b) Time management	“Of course, I am relatively flexible in planning my time, unless I am working on a specific project. In this case, a customer order definitely has priority but I love dividing my time freely, taking up and further developing new thoughts, discussing with colleagues or customers without having this terrible time pressure of not having to think things through to the end and nevertheless having to deliver results. I like it. The degree of freedom, of course.” (*male, 47 years, 12 years solo self-employed*)
	(c) Decision latitude	“Customers who give me the choice of carrying out the project the way I want to. Decisions are up to me and I am the expert within a given framework. Then I can develop freely, that’s what I like a lot.” (*female, 59 years, 18 years solo self-employed*)
(2) Task responsibility	(a) Task completeness	“You basically have a positive feeling when purchasing, planning, implementing und finalizing.” (*male, 55 years, 30 years solo self-employed*)
	(b) Diversity/variety	“A really large network of different people obviously connected by a different level of intensity and density. I met and argued with different people, ranging from small individual entrepreneurs to agencies and international top managers of large corporations – that is what diversity means to me. In another context, I would not have been able to experience all this.” (*male, 61 years, 24 years solo self-employed*)
(3) Responsibility for personal success	(a) Sense of achievement (quality of work)	“I am doing a good thing with my educational work and that’s a good feeling.” (*female, 32 years, 4 years solo self-employed*)
	(b) Appreciation/respect	“Yes, as I said at the beginning, 100% recognition. I worked on a project and I completed the project. I somewhat don’t have to share it with anybody else. Indeed, I alone have to accept criticism, but, thanks god, compliments prevail. I am solely praised for my work – and this is pretty cool.” (*male, 53 years, 17 years solo self-employed*)
	(c) Good cooperation with customers (clients)	“Participants and customers who are solution-oriented involved as well as just motivated people and those who are dissidents. This kind of people might be a burden at work. People who do not really feel like cooperating and, at the same time, relieving if customers just like cooperating. And that’s the main point, I guess, having deliberately cooperating customers.” (*female, 25 years, 0.5 years solo self-employed*)
	(d) Meaningfulness (usefulness)	“A media business administrator recently said: ‘This is fascinating. Exactly the problem we worked on only 1 week later arose in our company and it was so good that I was able to explain how this works.’ These incidents are certainly extremely positive.” (*male, 59 years, 14 years solo self-employed*)
	(e) Synergies related to multiple job holding	“It certainly also supports me in relation to my work at hospital, where I am not always entitled to have a 100% say.” (*female, 47 years, 25 years solo self-employed*)
	(f) Balance of effort and reward	“A customer saying: ‘We really worked intensively on this project, my expectations were exceeded.’ is a relieving factor, of course. That’s great, of course. Getting paid adequately and achieving a turnover represents a relieving factor as well.” (*male, 47 years, 12 years solo self-employed*)
	(g) Learning and development options (further development)	“Personal development definitely has a decisive influence on the development of my own personality. This is worth its weight in gold. No matter if business turns up or down…the way I have changed skin like an onion within the last few years, I could give myself a slap on the shoulder, I just think that’s good.” (*male, 47 years, 12 years solo self-employed*)
(4) Personal economic success	(a) Income	“You know exactly that the X Euro you charged per hour will be yours in the end and that there is no other person saying: ‘Here a few percent.’ Ok, if you work in the service sector you are not paid a commission, but then I inform people on my hourly rate before and they have to count it up - which is normally no problem.” (*male, 51 years, 18 years solo self-employed*)
	(b) Follow-up orders (security)	“Office working hours just like today definitively represent a positive factor since you get a feedback and incoming orders. This is motivating and just a pleasant matter.” *(male, 58 years, 2 years solo self-employed*)
	(c) Building up of financial reserves/growth	“I built a house for myself and afterward built up the company in the industrial area, bought a bigger property, built a warehouse and, most importantly, I had industrial representations, i.e., from company L. or attic stairs from company R. and these roller shutter boxes I built in 1960.” (*male, 84 years, 51 years solo self-employed*)
(5) Sole design of interaction in social structures – social resources	(a) Family support	“What I mentioned before, the tasks my husband kindly takes over for me. Economic and accounting matters in particular and any internet related issues. My husband even installed a newsletter for me. I would not have been capable to get this off the ground all by myself. This is exactly where he really perfectly completes me. Otherwise I would fail. Without him at my side, I would have thought about starting up my own business in the first place and if, from the beginning, he wouldn’t had said: ‘I will help you, I will do it.’ (*female, 59 years, 1 year solo self-employed*)”
	(b) Support from colleagues	“Quality assurance in consulting actually plays a role. Intervention, supervision…Interaction and building up a room for your own questions. These are resonances…One of the reasons for this network, each of them with a personal and individual supervision.” (*male, 59 years, 20 years solo self-employed*)
	(c) Support from suppliers/external workers (interfaces)	“Where good preparatory work has been done, let’s put it this way, by the industry or the companies themselves having preset parameters and you know exactly: ‘Ok, this is the right person, you have to go there.’ That’s a positive aspect, that’s easy.” (*male, 58 years, 2 years solo self-employed*)
	(d) Social independence	“First of all, I realized that, of course, I am not responsible for other people. In hospital, for example, the quality of training was really bad. In my last position as assistant medical director, there was a time when many partly poorly trained assistant physicians came from Eastern regions. That was very stressful since, in the end, you were responsible for what they did in hospital. And that was really a tough affair.” (*male, 54 years, 11 years solo self-employed*)

Across the interviews, *autonomy* turned out to be the most significant resource. Autonomy refers to the degree of freedom people experience in their work, i.e., if they are free to decide how they want to accomplish a certain task and are not getting precise instructions on how the task is to be handled. Autonomy is, according to self-determination theory (Deci and Ryan, 1985, 2000), one of the basic human needs that has to be satisfied. To have control over one’s own working situation has been frequently shown to be a resource. It has direct effects on the well-being of the paid employed as well as indirect effects through diminishing the impact of work-related stressors (Zapf and Semmer, 2004; Sonnentag and Frese, 2012). In solo self-employment, autonomy is an inherent part of product and costumer decisions (1a; i.e., the decision of what and with whom to work), time management (1b; i.e., the decision of when to work) as well as a general decision latitude (1c; i.e., the decision of how to work). In contrast to employer entrepreneurs, this autonomy solely focuses on oneself as the requirements and needs of employees do not have to be taken into consideration. As stated by this interviewee: “I do not have to pay anyone more. That’s a financial advantage increasing my flexibility.”

Concerning sole *task responsibility*, it becomes evident that tasks have to be considered comprehensively and, according to action regulation theory (Hacker, 2003), should (2a) range from the processes of goal orientation, over planning, selection of necessary means as well as executing to inspection. This indicates that the meaning of the task, as well as the task feedback, becomes attributable to the person promoting well-being. Moreover, task variety (2b) decreases the risk of unilateral strain and promotes diverse knowledge, skills, and abilities.

*Responsibility for personal success* is derived from various resources for small business owners which are already known quantities from research with paid employed people. Its unique nature is caused by the quality of sole responsibility. This refers to one’s own actions being traced back to oneself, and the strengthening of self-esteem through that process (Semmer et al., 2007). As a protective mechanism subjective success experiences, i.e., the sense of achievement (3a) promote well-being, health and recovery and vice versa. Simultaneously, perceived personal success lessens potential health impairments (Grebner et al., 2010). Appreciation and respect (3b) foster self-esteem and promote health (Semmer et al., 2007). A satisfying cooperation with clients or customers (3c) reinforces the sense of belongingness and perceived social support (Semmer et al., 2007) which is essential for the solo self-employed as it eases goal fulfilment and secures follow-up orders. Meaningfulness (3d) means that the person acknowledges the benefit of his or her product or service and attributes it with a societal value (Hacker, 2003; Zapf and Semmer, 2004). Being a central part of the job characteristics model of Hackman and Oldham (1976), meaningfulness has been shown to be related to, among other things, increased intrinsic motivation (Fried and Ferris, 1987). For those solo self-employed who execute two or more jobs (multiple job holders; Kottwitz et al., 2017), synergies through higher degrees of freedom in solo self-employment compensate the constraints of other employment forms (3e). A balance between efforts and rewards should be established in terms of professional gratuity (Siegrist, 2000; 3e). Learning and development options (3f), which result from coping with challenging tasks that broaden the existing skills, promote mental flexibility and sustain one’s professional qualification (Hackman and Oldham, 1976).

In terms of resources, the sole responsibility of the worker for the *personal economic success* was further evaluated to be relevant. This includes securing the recent income (4a), the guarantee of follow-up orders in the short-run (4b) as well as the opportunity for savings (4c; building up financial reserves) in case of unfavorable times and for retirement.

Compared to the stressors in solo self-employment, the last resource refers to the social environment labeled as *sole design of interaction in social structures*. Besides family support (5a) indicating that the family partly takes on such tasks that co-workers from paid employment would have done to provide support from colleagues (5b) was reported including sometimes the establishment of large social networks. A productive collaboration with suppliers (5c; support from suppliers/external workers) was regarded to be relieving. Lastly, social independence (5d), i.e., being neither responsible for subordinates nor having to report to superiors, was regarded as an unburdening resource.

### The Role of Micro and Macro Factors for Shaping the Work Situation

To answer our third research question, and to complete our work psychological stress model for solo self-employed individuals, we explored the role of personality as a micro aspect as well as the market situation as a macro aspect. We already know from studies with dependent employees that a poor economic situation can have a negative impact on the situation of employment (Bispinck et al., 2010). Also, the resources and stressors derived from solo self-employment are shaped by the framing conditions of the market in which the product or services are offered and indirectly affect well-being. Moreover, the motives of the choice of this type of employment play a role: these motives can operate either as individual resources for driving an entrepreneurial life style, fostering resilience and helping to deal with potential obstacles through entrepreneurial self-efficacy, or they are an indicator of individual vulnerabilities when the executed type of employment does not match with the preferred one.

Entry into self-employment can be motivated by push or pull factors (Nabi et al., 2015; Patrick et al., 2016). Push factors are defined as factors that lead to escaping an adverse situation, e.g., unemployment or unsatisfying job conditions (Moore and Mueller, 2002; Saridakis et al., 2014). In contrast, pull factors refer to positively evaluated aspects of an entrepreneurial career path containing, for example, expectations of autonomy regarding timing, implementation of one’s own ideas, as well as higher income (Wang et al., 2012; Kolvereid, 2016). In the next chapters, we first describe the macro factors and how they are perceived by small business owners. Finally, we will then summarize the role of individual differences regarding the interviewees’ motives for going into solo self-employment.

#### Macro Effects: Framing Conditions by the Market

The market and product context contains factors such as order situation, competitive pressure (i.e., rivalry among existing competitors), or regulations which are valid for specific products/services (see, e.g., Porter, 2008). Obviously, solo self-employed work has determined itself by the rules of the market which sets the framing conditions for unfolding or limiting autonomy. In the interviews, only demands or stressors, but no resources, were surprisingly named.

The interviews suggest a close link between autonomy and the market and product context: On the one hand, autonomy comes along as a resource for selecting the product (and in that the task) and the market that has to be acquired. On the other hand, the market itself settles the boundary conditions and determines the level of autonomy. When it comes to the perceptions of the role of the market, however, the solo self-employed reflected on it as a boundary factor for their autonomy. Details can be found in Table 4, where the categories and sub-categories of the interviewees with added sample phrases for each sub-category are illustrated.

**TABLE 4:** Stress factors in the market and product context.

**Category**	**Sub-category**	**Example**
(1) Limitation in the decision-making process	(a) Market	“For a very long time, actually for the longest period of time of my self-employment, it didn’t play a role at all. Only for a few years, I would say since the financial crisis in 2008/2009. Customers are financial services, insurance companies and building societies, which are the most shaken groups on the market. Within the course of the last three years, I cancelled 90% of the counseling budget of my three biggest customers! And afterward they are a flypaper on the market.” (*male, 59 years, 20 years solo self-employed*)
	(b) Customer	“I am flexible in my work planning. Sure, the customer has to be fine with it, but in general, he orders something from me because he, let’s say, knows my signature.” (*male, 47 years, 1,5 years solo self-employed*)
	(c) Competition	“To be stuck in administration and billing related matters.” (*male, 40 years, 8 years solo self-employed*)
(2) Limitation in flexibility	(a) Customer	“I have now slightly adapted my program for this year. Last year, I offered walks during the week and finally realized that they were not well booked since most customers preferred walks on the week-end.” (*female, 59 years, 1 year solo self-employed*)
	(b) Knock-on effect	“The disadvantage is that if I work less I get fewer orders, if I work a lot, I get a lot of orders. This is something, which will probably be asked more often. That’s the biggest problem of self-employment. If I say that I would like to work a bit less, I immediately get less orders the following year.” (*female, 56 years, 30 years solo self-employed*)
(3) Dependence	(a) Local conditions	“The catchment area comprises almost 100.000 less people than in G. In Germany, M. is the city with the most expensive rents and students have less money. As a result, students spent less money for parties, thus club owners earn less money and pay DJs less money who, in return, are to pay their employees a minimum wage. Therefore, we have less money than a city like F. or G. That means it has something to do with where I play music.” (*male, 32 years, 7.50 years solo self-employed*)
	(b) Temporal conditions	“Temporal conditions in relation to holidays. You just don’t have holidays or rather only the holiday you pay yourself. That means you don’t have the safety of ‘I am continuously payed even if in August there won’t be any courses because of my holidays.’ These are company holidays – which don’t apply to me. I must plan completely differently. I must split costs accordingly for the entire period. That’s a second disadvantage.” (*male, 59 years, 14 years solo self-employed*)
	(c) Coordination with colleagues/suppliers/external workers	“There are of course situations in which I enter into an exchange with people and I surely face certain dependencies as regards termination, i.e., when we work together in a project in which I am certainly not solely involved and make arrangements with other people. In this case, it might sometimes be a stress factor, if I say ‘Okay, I have to discuss this with somebody,’ or ‘We have to find a date,’ or ‘I have to check something.’ But what I generally consider positively is the fact that I am not alone.” (*female, 25 years, 0.5 years solo self-employed*)
	(d) External factors	“One stress factor is my dependence on the weather. This is really stressful for me, since, for example on weekends, when I know I have to complete certain tasks outside, I am already sitting there checking what the weather will look like. This is stressful.” (*male, 53 years, 21 years solo self-employed*)

Overall, the market and product contexts cause a *limitation in the decision-making processes*. The market (1a) determines which products and services can and cannot be sold and at what point in time. It also sets the potential access to clients or customers (1b) who have an impact on the design of the product or service as the small business owners must align their supply to their customers’ demands. In some fields, external regulations (e.g., rules by the Association of Statutory Health Insurance Physicians) or closeness to other players in the market that drive competition (1c) determine the types of products or services, their quantity, and way of being offered and sold.

Also, there is a *limitation in flexibility* caused by the market. The customers (2a) determine the time frame of the order execution in certain ways. Knock-on effects (2b) in relation to one’s own marketability were reported; withdrawal from the market leads to secondary costs.

Finally, diverse *dependencies* emerged. These contained local conditions (3a) such as the necessity to move to the customers and temporal conditions, (3b) and the time when the market is open for products and services. Moreover, coordination requirements (3c) with colleagues, suppliers, or external workers and other external factors (3d) being out of the control of the small business owners were perceived as constraining to one’s independence.

#### Micro Effects: Personality, Motives, and (Fulfilment of) Goals

While the market serves as an external macro factor, internal micro aspects also have to be considered as inter-individual differences determine the perception of a situation and their ability to cope with it (Lazarus and Folkman, 1984; Zapf and Semmer, 2004). Based on Holland’s theory of vocational personalities (Holland, 1996, 1997), individuals choose work environments as a result of many different factors. These include their attitudes, values, abilities, personality, and job characteristics, as well as factors relating to organizational structure and culture (Van Vianen, 2000). When it comes to health and well-being, however, not only are motives and goals important, but the fit of motives and goals to their respective working conditions or job characteristics are also particularly important. Research from the field of person-environment fit indicates that career productivity is best when there is a good fit, which increases the likelihood of success and satisfaction (Holland, 1996, 1997).

The interviewees were asked why they wanted to become solo self-employed from the start and what their goals were then. Table 5 provides the five broad categories derived from the interviews and the sub-categories, with sample phrases for each sub-category. Notably, several parallels to the resources provided by those engaged in solo self-employment (see, Table 3) were found.

**TABLE 5:** Motives for choice of solo self-employment.

**Category**	**Sub-category**	**Example**
(1) Self-fulfilment	(a) Thematic interests (product decision)	“I didn’t aim at solo self-employment. You don’t have a big choice or a variety of possibilities. If, as a photographer, you don’t want to be employed in a photo studio where you have to take pictures of sandwiches all day, you become a freelance photographer. It’s the same with graphic designers.” (*male, 47 years, 1,5 years solo self-employed*)
	(b) Solo self-employment as sole employment form for selected profession	“First of all, there was no ‘why’ since in my sector, there is no other possibility. As a dancer, dance educator and fitness trainer you are always solo self-employed. You didn’t have a choice.” (*female, 27 years, 7 years solo self-employed*)
	(c) Freedom of choice regarding the execution of contract (customer decision)	“I really wanted to get things moving for customers with a certain strategic or knowledge interest. I was originally employed in a company structure in which you sometimes asked yourself whether you are really needed or not. The question is whether you always want to ask yourself why you are doing a certain job. Insofar, I like working together with customers who, of course, have a concrete concern they are willing to pay for. Thus, this is about real exchange and interest and not only a formal and functional interest. This is at least what I would like to think. Working together with people on a relevant issue.” (*male, 47 years, 12 years solo self-employed*)
	(d) Autonomy regarding method	“If I think that something doesn’t work as successfully as it should, I want to be able to intervene. If people don’t listen to my advice I want to be free to decide that this is their decision which, however, I don’t support and consequently leave them alone.” (*male, 51 years, 18 years solo self-employed*)
	(e) Autonomy regarding time	“It was the flexibility to do things with R. and to decide solely when I would go to France in order to visit my family that confirmed my decision of self-employment, i.e., not to work in a wine shop. I don’t want to be limited in actions, I cannot image.” (*female, 59 years, 18 years solo self-employed*)
	(f) Task closure	“I aimed to operate in a holistic work enabling me to take care of a women during pregnancy, at birth and even afterward. This is actually the ideal image of my job.” (*female, 47 years, 25 years solo self-employed*)
	(g) Variety	“…variety. I have the feeling that my job is just varied.” (female, 55 years, 25 years solo self-employed)
(2) Career aspects	(a) Own business	“At the beginning I said that I would build up a joiner’s workshop and that 1 day they will have to carry me out of it feet first and that was it.” (*male, 53 years, 17 years solo self-employed*)
	(b) Building up something (growth, sustainability)	“That corresponds to what I said before. In principle, I aimed at setting up a more classical consultancy with a pyramid structure of chief advisors and other consultants, assistants and trainees including a solid secretarial structure, local organization, professional marketing and advertising strategy and so on.” (*male, 57 years, 32 years solo self-employed*)
	(c) Entry into a sector (pull-motivation)	“With the goal in mind what motivated me or the fast entry into a sector which otherwise I would not have been able to get into.” (*female, 25 years, 0.5 years solo self-employed*)
	(d) Exit from a sector (push-motivation)	“I had simply imagined continuing to work in my previous profession until retirement. I just didn’t find that tempting at all.” (*female, 59 years, 1 year solo self-employed*)
(3) Job security	(a) Avoidance of unemployment	“I actually imagined being active as independent works council chairman and lecturer at the same time, of course, until retirement. That was my plan until Hartz IV** was introduced. Then suddenly I was sitting there. I have pondered for a few months or almost half a year. Should I look for a job somewhere else, but a job as what? Where? How?” (*male, 59 years, 14 years solo self-employed*)
	(b) Insecurity regarding qualification	“When I was a heating engineer, I was self-employed as well. Then I gave up. Afterward I had worked as employee for a long time and now again. Since the company I last worked for became insolvent, I applied for a job somewhere else where people told me that I was overqualified. Then I said to myself: “Ok, I will start my own business.” (*male, 61 years, 4 years solo self-employed*)
	(c) Insecurity regarding age	“I became unemployed but due to my experience not everybody disposes of, I thought that I would certainly find another job some time. However, it always came down to age being a point where most people said: ‘No,’ you are too old for us; we are looking for younger people, if possible at the age of 35, with a degree and 20 years of professional experience.” (*male, 58 years, 2 years solo self-employed*)
(4) Income	(a) Better income (improvement)	“There were two jobs for me on the job market. I could start somewhere for 1,200 euros what was not actually a salary I was looking for, because for 1,200 Euro net, I would have said soon: ‘I don’t have to get up in the morning.’ That is not interesting for me. Especially since occurring costs or costs, which might occur for the employer, would be passed on to the agent, i.e., paper, etc. I would have had to do everything myself and 1,200 euro is by far not enough.” (*male, 58 years, 2 years solo self-employed*)
	(b) Making profit	“One goal was definitely always a financial goal since the potential of earning money in this sector was very, very high, at least 12 years ago. So that’s the financial issue.” (*male, 47 years, 12 years solo self-employed*)
	(c) Adequate income (gratification)	“I finally wanted to be paid according to my educational level because at one point I just became too expensive for my former employer. Or rather they didn’t want to accept my salary claim.” (*male, 40 years, 8 years solo self-employed*)
	(d) Financial independence	“That was first and foremost financial independence, as I described before. I have always been annoyed that people benefited from my performance, whether it was the master, manager or director in F.” (*male, 84 years, 51 years solo self-employed*)
(5) Compatibility with private life	(a) Reduction of working time	“At that time, one goal was definitely to work less since my weekly working time amounted to 60−80 h and I thought that even from a health-related aspect I would not be able to stand this pace if I stay in this job. Although, I actually would have had, from a purely formal point of view, very good prospects in my former job.” (*male, 54 years, 11 years solo self-employed*)
	(b) Encouragement of personal balance	“A job offering a good balance between personal interests, free time and job engagement, involving pleasure and further development as well as working together with pleasant people.” (*male, 61 years, 24 years solo self-employed*)
	(c) Fulfilment of family/private responsibilities	“I decided to stay at home with my children and that was most compatible with self-employment. That was actually the main reason.” (*male, 53 years, 21 years solo self-employed*)

**Hartz IV, a set of recommendations presented by a commission in 2002 under the lead of Peter Hartz. In the fourth stage of the proposed reform of the German labor market, the former unemployment benefit for the long-term unemployed and welfare benefits were merged, so that both are roughly at the lower level of the former social assistance.

Most importantly, *self-fulfilment* was named. This was relevant to thematic products and services (1a; thematic interests) or professional decisions (1b; sole employment form for selected profession) as well as the freedom of choice regarding contracts and customers (1c). Moreover, the small business owners preferred to have autonomy regarding the methodology they used (1d; the how) as well as the time they work (1e; the when). Finally, to have control when it comes to task closure (1f) and to have a large variety of tasks (1g) were motives to follow this career path.

Next, the solo self-employed described *career aspects* as guiding motives for the choice of this employment type. Some reported that they liked the idea of having their own business (2a) which may grow eventually (2b; building up something). Also push and pull factors played a role as people entered a sector (2c; pull motivation). These included appreciated aspects of an entrepreneurial career path or exiting from a sector (2d; push motivation) because of unsatisfying working conditions in paid employment (e.g., Moore and Mueller, 2002; Saridakis et al., 2014).

In times of high uncertainty and with atypical employment on the rise (Selenko et al., 2018), the small business owners also reported having chosen their employment type to keep *job security*. This contains statements that indicated an avoidance of unemployment (3a) as well as of failed attempts to find paid employment because of one’s qualifications (3b) or age (3c).

In addition, *income* was a central parameter of objective career success (Gunz and Heslin, 2005) and was regarded to be a prime motive. Specifically, the prospect of a better income (4a) as compared to the situation in paid employment as well as making profit (4b) was named. Additionally, the interviewees aimed at achieving a balanced fit between effort and reward (4c; adequate income; Siegrist, 2000) through solo self-employment. Finally, some were motivated by having financial independence (4d) from others.

The last reason was that a solo self-employed job offered a better *compatibility with one’s private life*. Here, the opportunity to reduce working time (5a), the encouragement of compensation from work (5b; i.e., promoting a personal balance) as well as an enabling of the fulfilment of family or private responsibilities seemed to be the driver to choosing the entrepreneurial career path.

## Discussion

### Interpretation of Results

The aim of the present study is to gain an in-depth understanding of how small business owners in Germany perceive their working situation, considering stressors and resources as well as motives and the surrounding market conditions. Using expert interviews (Bogner et al., 2009), we aimed to answer three research questions: first, to get an understanding of the well-being of solo self-employed people by reflecting their options to recover. Second, we aimed to explore the stress or-strain relationship building on ideas of the transactional stress theory by Lazarus and Folkman (1984) in order to develop a work-psychological stress model for the solo self-employed. Third, we further analyzed how factors within the person (e.g., micro level, personality) and in the market (macro level) add to understanding the causes of mental well-being or strain.

Regarding the first research question, we found that well-being is an important issue to consider. For example, the small business owners in our sample demonstrated signs of presenteeism (i.e., working in case of illness; Johns, 2010). This indicates that when people begin to go into business they have other priorities than protecting their health. A high risk of self-exploitation seems to be inherent within an entrepreneurial career path. In pursuing desired success, they overcommit to the business at the expense of their health and family work balance (McDowell et al., 2019). As there are no protections by labor protection laws regulating their working time or time for recovery this risk is hard to control from the outside. Moreover, the solo self-employed have the autonomy to regulate their work patterns, and therefore turn into their own abusers of their right to recovery.

In considering the second research question, we found some stressors and resources which were comparable to other jobs but having sole responsibility for each and every part of the working conditions makes them especially vital. In studies with dependent employees, it became clear that too much responsibility and a role overload is perceived as a stressor that is associated with health impairments (e.g., Kivimäki et al., 2002; Vanishree, 2014). Solo self-employed people also report various stressors (e.g., charge of all tasks, lack of time or handling of difficult customers) due to having sole responsibility. At the same time, they also view responsibility as a resource and opportunity for self-realization. Self-realization is in turn positively related to health. Thus, the double role of responsibility in the work of the solo self-employed is unique.

When trying to answer the third and last research question, we found that aspects on the micro and macro level should not be neglected when looking into the stress−strain relationship of solo self-employed people. For the macro level, the market conditions strongly determine how work can be created considering both stressors and resources as it constrains the flexibility and affects health and strain indirectly. Moreover, high dependency from suppliers, customers, and colleagues reduced autonomy. This turns responsibility into a stressor and decreases well-being. Moreover, the macro level is interrelated with the micro level by shaping working conditions. They determine if a person-environment fit (Caplan, 1987) can be achieved. Perceived congruence between personal and work environment factors results in more readiness for a given career path and a higher well-being (Jiang and Jiang, 2015). Micro aspects such as motives, interests, goals, abilities, or personality factors are relevant for the readiness to go into solo self-employment (Baluku et al., 2018c). It is also relevant for selecting the specific field to which the product or service belongs to and in which market (Holland, 1996, 1997). Finally, it is relevant when evaluating, framing, and coping with job characteristics (Lazarus and Folkman, 1984; Lazarus, 1999).

Finally, our developed stress model highlights the specific conditions of solo self-employed people for whom resources and stressors are more closely linked than for paid employed individuals or employer entrepreneurs, and for whom resources and stressors are equally determined by their sole responsibility. On the positive end, autonomy as an overarching framework, which is based on self-determination theory (Deci and Ryan, 1985, 2000), is one of the basic human needs that can be perfectly satisfied through solo self-employment if people prefer autonomous work and sole responsibility (*micro aspects*). To achieve this, they create their working conditions in a way that autonomy can count as a work-related resource and not so much as a stressor (*meso aspects*), and when the macro aspects of the market allow flexibility (*macro aspects*). However, on the negative end, solo self-employment is associated with high insecurity caused by uncertain market conditions, a high dependency on customers or suppliers, a low person-environment fit by being pushed to this career path, or by dealing with adverse working conditions and having no recovery opportunities. This type of employment would be bad for a person’s well-being.

### Strengths and Limitations

To the best of our knowledge, this is the first study examining the interplay of stressors and resources shaped by market conditions and the personal motives of solo self-employed people. While there is a lack of research overall with this specific group, it is clearly a strength of our study to explore their working conditions in detail, as this group is less secured by support from specific unions or other representatives as the group itself is extremely diverse. In our study, we interviewed a cleaning woman as well as a physician. It might still be difficult to reflect the broadness of this employment type.

While we used a representative study for our typology to build a broad picture on solo self-employed people from various sectors, the economic situation and demographic data such as age and gender might still not reflect people at extreme ends that are not contained in the representative data. For the people on the very prestigious end, they might not have the time or feel the need to take part in the study – so they might not be reflected in our sample of the solo self-employed. However, it can be assumed that these people have advantageous working conditions. The lack of people on the precarious end might be more problematic as they might be involved in precarious types of solo self-employment. To be part of the BIBB/BAuA employment survey of the working population on qualification and working conditions in Germany, one must be fluently able to speak the German language on the phone. Hence, there might be small business owners that never entered into the data pool as they were not German speaking. Also, as our interviews were conducted in German, it would have been difficult to interview such solo self-employed people who were not able to reflect on their situation in German. This must be considered when generalizing the data.

### Theoretical Contribution and Practical Implications

Although there is growing literature on individual psychological factors that determine entrepreneurial intentions, persistence, and success (e.g., Baluku et al., 2018b), little is known about how the job of being a small business owner looks like from a work-psychological perspective. Our study adds to the limited research on the specific working conditions of small business owners (in our case: solo self-employed). A significant group of people, not only in Germany, are engaged in solo self-employment. Moreover, there is a paucity of research focusing on the realities of their work and working conditions, their lived experiences of success and constraints, and how these affect their other domains of life. Our study, therefore, generally brings knowledge to some important insights that can stimulate research into the different issues involved in solo self-employment that affect the lives of people in this type of employment.

Arguably, it has been observed that entry into self-employment tends to increase in the face of changing dynamics in labor situations, such as limited opportunities for salaried positions (Rissman, 2003; Falter, 2005). While the German labor market still offers a variety of jobs in dependent employment, there are occupational fields (e.g., journalism, nanny) where solo self-employment is quite common. Yet, not all small business owners voluntarily chose and follow this career path as shown in our interviews. Particularly for those being pushed into self-employment, they were not attracted (“pulled”) by its autonomy and decision latitude. Hence, they might face the downside of this employment type to a greater degree as they might have the same amount (or even more) of job stressors (as high economic insecurity, dependency/conflicts with clients) but will not perceive the inherent employment opportunities of autonomy as a resource (in contrast to those who chose this career path to fulfill their need for autonomy). Future studies should further look into the impact of voluntariness in the long run and try to uncover if solo self-employed workers get used to the (once “unwanted”) entrepreneurial role if they succeed with their business and eventually start to like this employment type with its autonomy and sole responsibility. This would offer more job resources to buffer strain and sustain health. They could also possibly continue to “suffer” under this role as it does not match their preferences (“person-career-fit”), resulting in consequences for their health and well-being.

Moreover, we conducted interviews in a developed country where the pressure to become an entrepreneur is comparably low than in less developed countries, where entrepreneurship might be the only viable option (e.g., Baluku et al., 2019, 2020). Hence, the “push” to start a business might increase for people in developing countries. Financial security and social safety could be even lower there, resulting in poorer working conditions and health risks. Yet, it could also be the case that those small business owners have levels of higher resilience. Further research should take work situations, resources, stress, and strains from a cross-country and cross-cultural perspective into account and to add further macro factors – such as culture, economic conditions, or the social safety net of a country – to our developed model.

Notably, our results also have implications on dependent employees. With increasing flexibility, more dependent employees are also working in a highly marketable way and have to organize their work themselves outside of company structures. The current changes in work forms also require a strengthening of the health competence of employees, their participation in the design of work processes, and the support of non-business actors (e. g. health insurance companies).

Our findings indicate that some self-employed individuals have trouble with the time required for recovery from work related fatigue and from sickness. The question that emerges from this finding is what can be done to support solo self-employed individuals to have adequate time and personal resources for recovery despite the pressure that work places on their time. Recovery being a relevant issue also became apparent when questioning the interviewees about their wishes and further suggestions regarding measures of occupational safety and health (OSH). As one small business owner stated, “I would simply say to take a cure somewhere means for me being out of professional life for 3 weeks. It does not work. It’s fatal, that does not fit.” Limitations in structural opportunities (e.g., participating in a back-training course was impossible due to time reasons), lack of information on OSH (e.g., how often one should take breaks) or no adequate support for the self-employed at all (e.g., health insurances should offer courses on occupational safety and health protection) were reported.

Similarly, there are aspects in the work context and personal motives that are potential stimulators of strain and stress. Hence the question is how solo self-employed individuals can be supported to cope with the demands exerted by professional and personal goals. An important insight here can be derived from positive psychology. Given that psychological resources and capital such as self-efficacy and resilience are reported to support the psychological health of entrepreneurs (Baron et al., 2016), it is important for the relevant authorities to develop interventions that support the development of psychological resources of small business owners.

## Data Availability Statement

The datasets generated for this study are available on request to the corresponding author.

## Ethics Statement

The studies involving human participants were reviewed and approved by Ethical committee of the Faculty of Psychology (Marburg). The patients/participants provided their written informed consent to participate in this study. Written informed consent was obtained from the individual(s) for the publication of any potentially identifiable data included in this article.

## Author Contributions

KO, LH, and MK collaborated in a project investigating the health of people in solo self-employment from which the data was collected. MK and KO developed the concept. KO, MB, and MK structured the ideas for this article. MK performed the analyses and reporting in German. KO wrote the first draft. All authors read and approved the final manuscript.

## Conflict of Interest

The authors declare that the research was conducted in the absence of any commercial or financial relationships that could be construed as a potential conflict of interest.
